# Characteristics of Venture Capital Network and Its Correlation with Regional Economy: Evidence from China

**DOI:** 10.1371/journal.pone.0137172

**Published:** 2015-09-04

**Authors:** Yonghong Jin, Qi Zhang, Lifei Shan, Sai-Ping Li

**Affiliations:** 1 School of Business, East-China University of Science and Technology, Shanghai, 200237, China; 2 Institute of Physics, Academia Sinica, Taipei, 11529, Taiwan; Universidad Rey Juan Carlos, SPAIN

## Abstract

Financial networks have been extensively studied as examples of real world complex networks. In this paper, we establish and study the network of venture capital (VC) firms in China. We compute and analyze the statistical properties of the network, including parameters such as degrees, mean lengths of the shortest paths, clustering coefficient and robustness. We further study the topology of the network and find that it has small-world behavior. A multiple linear regression model is introduced to study the relation between network parameters and major regional economic indices in China. From the result of regression, we find that, economic aggregate (including the total GDP, investment, consumption and net export), upgrade of industrial structure, employment and remuneration of a region are all positively correlated with the degree and the clustering coefficient of the VC sub-network of the region, which suggests that the development of the VC industry has substantial effects on regional economy in China.

## Introduction

In the past two decades, the Chinese market has been one of the fastest growing markets in the world. Since 2011, China has become the second largest economy in the world. Alongside with its booming economy, China opened the Shanghai Stock Exchange in December 1990 and the Shenzhen Stock Exchange in July 1991. Due to the stringent listing requirements, the two stock markets in China did not facilitate small and medium enterprises to raise capital until recently. Research showed that small and medium enterprise owners in China relied mainly on the financial support from their own savings and immediate family [1]. In developed economies, venture capital is an important alternative source of finance for small and medium enterprises, especially high-tech or growth-oriented small and medium enterprises. It offers potential for and challenges in financing the creation and development of entrepreneurial ventures, especially where alternative funding sources such as bank debt are problematical. Viewing this as a possible solution, the Chinese government attempted to promote venture capital to fill the SME finance gap from the 1980s, and the Chinese VC industry has grown significantly since the late 1990s. Being one of the fastest growing economies in the world, it is natural that China has attracted a lot of attention from overseas investors and a considerable amount of foreign capital. Of particular interest to overseas investors are venture capital investments in recent years.

Since more and more venture capitalists from developed nations are looking to developing economies for attractive investment opportunities and local entrepreneurs, a better understanding of the venture capital industry in those economies is necessary. Despite the dramatic growth of venture capital inflow into developing nations such as China and the substantial differences that exist between the venture capital industry in developed and developing economies, there are still relatively few studies to examine the economics of the venture capital industry in developing nations.

China’s venture capital industry had a slow growth in the 1980s and started to flourish in 1999 and 2000, a time period characterized by strong stock market performance and investor optimism. Since then, China represents one of the fastest growing markets for venture capital investment in the world. Venture capital investments in China come from different sources: government, state-owned enterprises, private firms, public companies, non-banking financial institutions, multinational corporations and foreign venture capital funds. VC investments in China also vary along several dimensions such as size, stage, voting control, duration, and location. It is also noted that a majority of VC investments are made in the Eastern and Southeastern industrialized coastal regions, which offer VC companies more opportunities. Indeed, about 90% of all VC firms that invest in China specialize in and seek out investments in the high-tech sector.

Venture capital investments involve a high level of risk, but on average also yield high returns. Given its present economic development, Western interest in China will likely continue for some time. Yet, entering the Chinese market is not straightforward and is a challenging task for investors. The different underlying structures of these capital markets imply that the differences between the Western VC markets and the emerging VC market in China remain large. Profit maximization, efficiency, and public information disclosure are important factors for Western investors. Venture capitalists in mature economies have found ways to reduce the risk associated with new venture investment. They do this by careful due diligence, providing management and personnel assistance, careful monitoring of the investment, and well-planned exit strategies in mature economies where the rule of law is well established for venture capitalists to reduce nonsystematic risk relatively easily. In contrast, personal relationships, networking, seniority and harmony are ranked highly in East Asia [2–3]. In particular, the Chinese culture places a large emphasis on the maintenance of connections or networking, under which harmony with and within organizations is frequently favored over information disclosure and shareholder rights [4]. In addition, the Chinese culture typically has a high tolerance for information asymmetry between the firm’s insiders and external investors as well as outside board members. It has been noted that cultural differences, corporate governance structures, a lack of appropriate exit strategies, and governmental intervention are important factors that set the markets apart and should be taken into consideration when making venture capital investments in China. In short, while there has been a dramatic growth in venture capital in China in recent years, the understanding of the industry remains insufficient and more research on this subject is necessary.

In recent years, complex network theory has been developed through the study of complex networks in natural and social fields [5–7], e.g., road map networks, food chain networks, brain neuron networks, voter networks and social influence networks. Most of these social, biological and technological networks have substantial non-trivial topological features which are categorized as complex networks. Complex networks have been extensively studied, and the results have been applied to many real world networks, such as biological nets and the World-Wide-Web (WWW) [5, 8–10]. Two fundamental topological properties of real complex networks have been paid much attention to, namely, the small-world and the scale-free properties. The small world structure of a network implies that the number of nodes increases exponentially as a function of the “diameter” of the network [11–14]. On the other hand, a scale-free network is a network of which the degree distribution follows a power law, at least asymptotically. Recent interest in scale-free networks was pioneered by Barabasi and his coworkers who studied the topology of the World Wide Web [6].

Recently, there also exist studies on the self-similarity [15] feature of a network, which is characterized by a fractal dimension [16–19]. The fractal nature of a self-similar network can be calculated by box-counting method [20]. Zhou et al. proposed a new box-counting method based on edge-covering, in which, the minimal number of boxes of size is obtained by the simulated annealing algorithm [21]. Different authors have proposed a wide range of alternative network coarse-graining techniques [22–25].

In the study of complex financial networks, Albert et al. studied the scale-free properties in stock market [8]. Kim et al. studied financial correlations [22]. Schweitzer et al. analyzed the new challenges of economic networks, and they treated the economy as a complex system which reflected dynamical interactions of a large number of different agents [26]. Kenett et al. used partial correlation network to study the dominant stocks which drive the correlations present among stocks traded in a stock market [27]. Zhang et al. studied the systemic risk and causality dynamics of the world international shipping market [28]. Tumminello et al. used a correlation matrix to define and obtain hierarchical trees, correlation based trees and networks of financial market [29]. Song et al. investigated the daily correlation present among market indices of stock exchanges located around the world in the time period January 1996 through July 2009, and discovered that the correlation among market indices presents both a fast and a slow dynamics [30].

There are papers exploring the relationship between complex financial networks and regional economies. Ausloos et al. represented a system by an evolving network, nodes being the GDP fluctuations (or countries) at different times [31]. Garlaschelli et al. presented an empirical analysis of the network formed by the trade relationships between all world countries, or the World Trade Web (WTW) [32]. Kronberger et al. briefly discussed the interaction network of macro-economic indicators and presented two identified models to approximate the help wanted index and the CPI inflation in the US [33].

In the field of venture capital investment, empirical studies have been carried out to investigate the bridging and clustering relations and it is found that, for example, the dense clustering of relations facilitates identity formation [34], coordination [35] and trust [36–38]. Hochberg et al. studied the venture capital networks of American venture capital funds between 1980 and 1999 and analyzed the characteristics of the network [39]. Zheng also studied venture capital networks and analyzed their prominence, range, brokerage, and cohesion, and concluded that corporate VC networks are not cohesive [40]. Mas et al. studied VC networks and found that they are not random, and the different assortativities (degree, spatial, industrial) are positive, suggesting that venture capitalists tend to co-invest with their peers [41].

Since there are differences between the operations of venture capital firms in China and the Western countries, it would be interesting to study VC networks in China and compare their differences. In this paper, we will study the complex network of venture capital firms in China. Venture capital firms face great risks and uncertainties in the investment process, and have to take actions to deal with them in a venture capital network. The formation of venture capital network, in a narrow sense, means that the firms which have common investees are connected. In this network, the venture capital firms contact and share resources with each other in many ways which reduce the costs of the firms in the network; and in a broad sense, it means that the firms from the same area and have common investees in the same industry are connected. The network would therefore be of great help to the venture capital firms and their investees in many aspects. Furthermore, as an organic composition of macro-economy of a country, the VC industry would likely be influenced by the progress of its macro-economy and the growth of venture capital investment of an area makes a great contribution to its local economy. In other words, there should exist correlations between the VC industry (or VC network) and the macro-economy of a region. We will also explore characteristics of China’s VC network and its correlations with the macro-economic indices of China in this paper. In particular, we would study the effect of the regional economy of China on the structure and properties of the Chinese VC network.

The paper is organized as follows. In Section 2, we define the VC network of the venture capital firms in China. Its statistical properties, namely degree, mean lengths of the shortest paths, clustering coefficient and robustness are calculated in Section 3. Section 4 analyzes the topological properties of the VC network. Section 5 discusses a multiple linear regression model on the relation between the statistical properties of the VC network and major economic indices of a region. Section 6 is the conclusion.

## The VC Network

We will present the VC network in China in this section. Our analysis is based on the listed companies from Chinese Growth Enterprise Market (GEM) and Chinese Small and Medium-sized Market (SME) in 2011. The empirical data come from Resset database and the prospectus of the companies which are listed in GEM and SME. We collect the data of venture capital firms investing the companies listed in GEM and SME. There were a total of 614 venture capital firms in 2011 as recorded by these databases.

Using the data above, we established the network of venture capital firms in China. It is a network that two venture capital firms connect to each other if they have at least one common investee.

Previous studies of venture capital networks are mostly based on the syndication [39, 42]. Our network here follows the same rule that links lie between syndical companies. Analyzing the network by location, we note that the links between developed areas of venture capital are denser than others.

**Fig 1:**
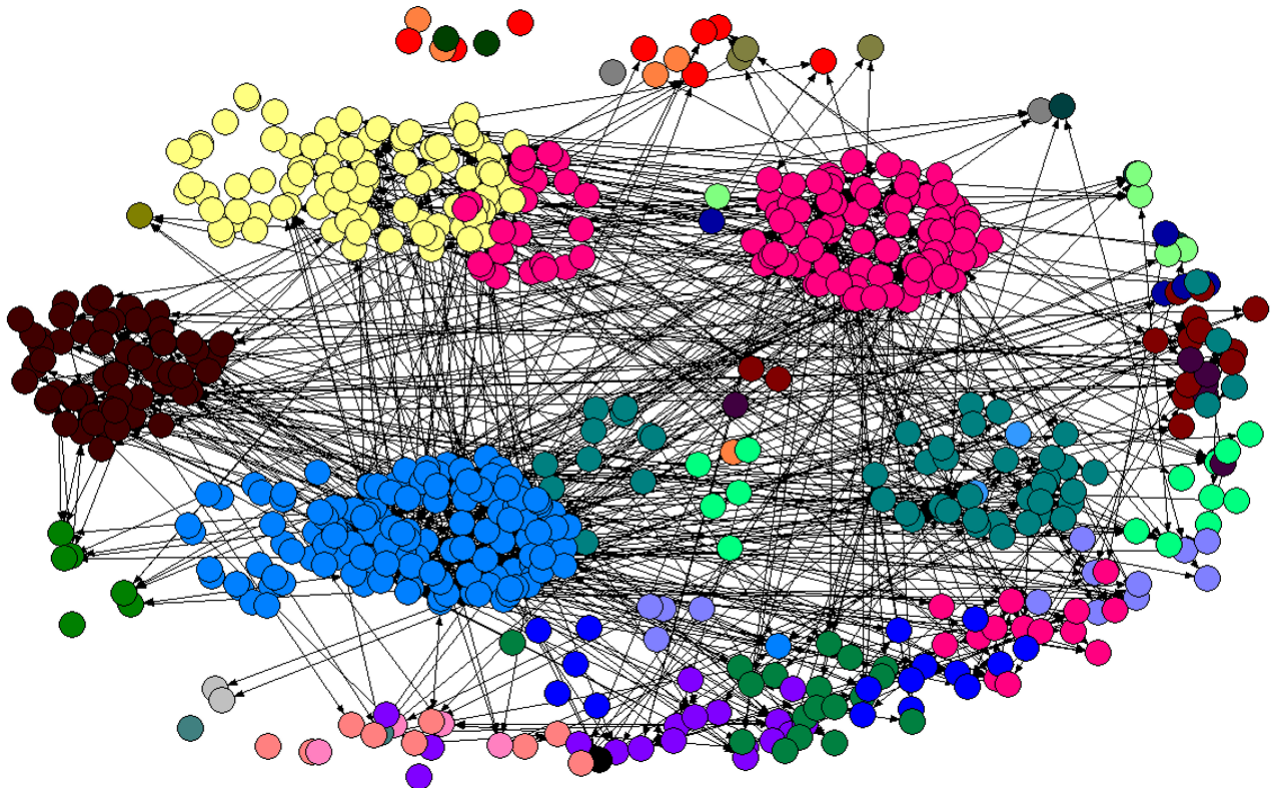
The VC Network of China. Nodes of the same color belong to the same province/district.

**Fig 2:**
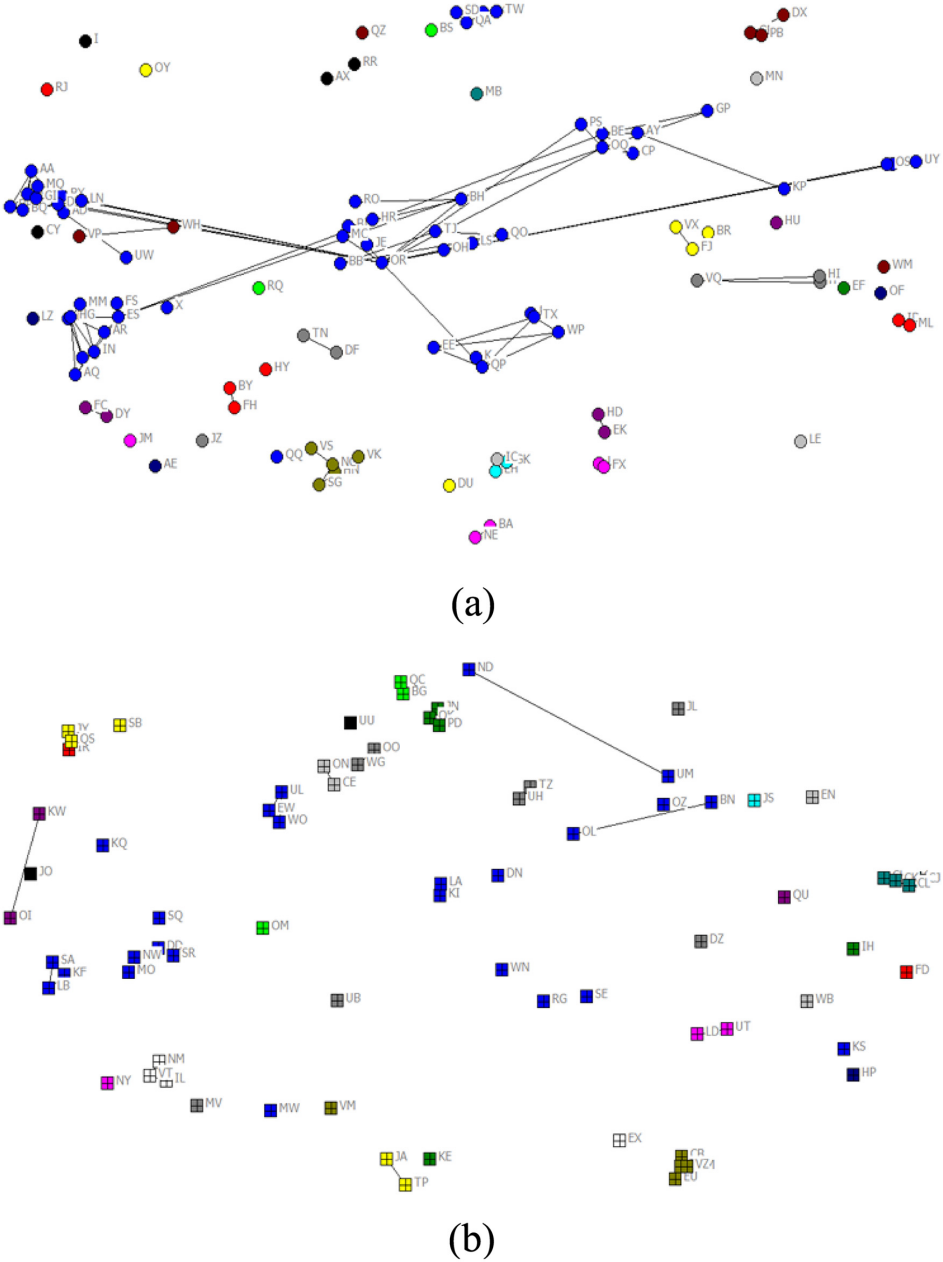
VC network of (a) Guangdong and, (b) Shanghai. We give an abbreviated name for each node in order to distinguish them, while the name of each node does not make sense.

Fig 1 is the VC network of China. Each node in the network denotes a venture capital firm while each line represents a link between two venture capital firms. Nodes with the same color are from the same province/district. One can see that there are clusters of nodes with the same color, meaning that some regions in China have a lot more venture capital firms than other regions. The reader is referred to Table 1 and Section 3 below for detailed discussion. Fig 2 shows the VC networks of (a) Guangdong and (b) Shanghai, which are two districts with largest degrees. From Fig 2, we see that the links within the same district is sparse, indicating that VC companies prefer to syndicate with partners from other districts.

**Table 1:** Total degree of each province/district in the VC network of China.

Province/district	Degree	Nodes	Province	Degree	Nodes
Anhui(AH)	11	9	Nei Monggol(NMG)	3	2
Beijing(BJ)	172	94	Ningxia(NX)	6	2
Fujian(FJ)	15	14	Shandong(SD)	41	18
Guangdong(GD)	385	139	Shanxi(SX)	4	2
Guangxi(GX)	9	4	Shanxi(Xian)(SXX)	9	11
Hainan(HN)	11	8	Shanghai(SH)	215	102
Hebei(HB)	0	1	Sichuan(SC)	23	16
Henan(HN)	29	13	Tianjin(TJ)	36	19
Heilongjiang(HLJ)	3	3	Foreign-owned	7	5
Hubei(HB)	54	14	Xizang(XZ)	1	1
Hunan(HN)	30	14	Hong Kong(HK)	20	6
Jilin(JL)	4	1	Xinjiang(XJ)	5	3
Jiangsu(JS)	143	56	Yunnan(YN)	0	2
Jiangxi(JX)	4	1	Zhejiang(ZJ)	98	46
Liaoning(LN)	5	3	Chongqing(CQ)	11	5

## Statistical and Topological Properties of the VC Network

In this section, we will study the statistical and topological properties of the Chinese VC network.

### Mean Shortest Path

**Fig 3:**
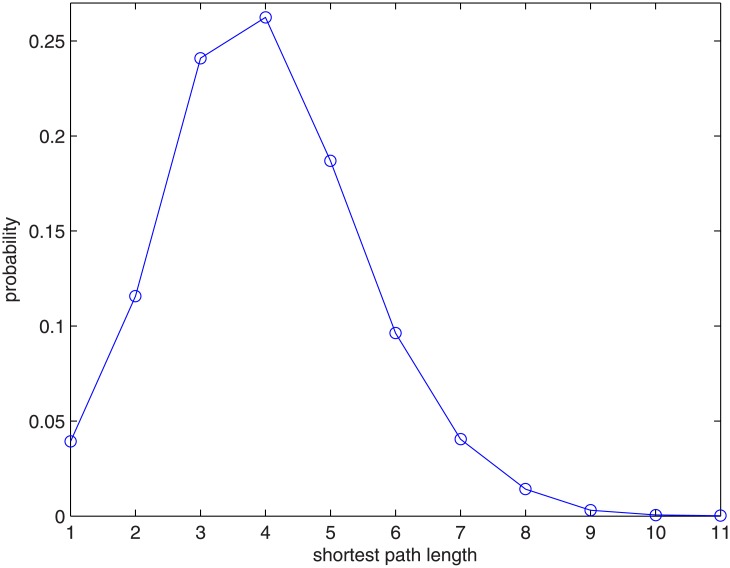
Probability density function of the shortest paths between two nodes of the Chinese VC network.

The mean shortest path of a network can be calculated as follows:

$$\bar{l}=\frac{2}{N(N-1)}\sum_{i\geq j}d_{ij},$$ (1)

where *d*_*ij*_ is the distance of the shortest path between nodes *i* and *j*. However, in many real world networks, the connectivity between two nodes cannot be guaranteed. We here denote the distance of the disconnected pairs as *INF*, and modify the formula [43] for the mean length of the shortest paths of a network as follows:

$$\bar{l}=\frac{1}{M}\sum_{\begin{array}{l} \begin{array}{cc} & i\geq j \end{array}\\ d_{ij}\neq INF \end{array}}d_{ij},$$ (2)

where *M* is the number of links with finite distances. The probability density function of the length of the shortest paths between two nodes of the VC network of China is shown in Fig 3 below. It is easy to see that most of the paths between two nodes in the network are 3 ~ 6 steps. The mean shortest path of the Chinese VC network can be calculated and is about 4.83.

### Degree

The degree of a node is the number of links that are associated with this node. It is one of the most important characteristics of a complex network. In general, the higher the degree is, the more important the node is.

**Fig 4:**
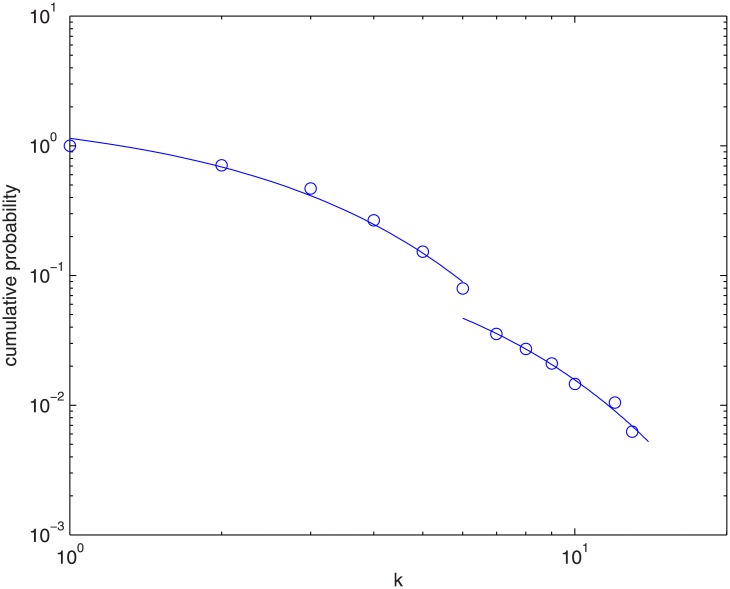
Cumulative degree distribution of the VC network in China.

Since our main purpose is to investigate the effect of the VC network on regional economy, we will here study venture capital firms in terms of districts, and evaluate the total degree of each province/district in the VC network instead of individual VC firm (node). One can check the degree distribution of the VC network in Fig 4 below. Table 1 is the regional statistics of venture capital firms. Most of the venture capital firms are located in Guangdong (GD), Beijing (BJ), Shanghai (SH), Zhejiang (ZJ) and Jiangsu (JS), where the regions are relatively developed and have rich capital and with a lot of economic activities going on. Companies there bear less cost and have greater competitiveness. On the other hand, there are districts where their firms have no links with others, which suggests that they have very low levels of economic activities. We will come back to this in Section 4.

### Clustering Coefficient

Clustering coefficient is used to describe the formation of small groups within a network. The average clustering coefficient *C* is a measure of the degree of nodes in a graph tending to cluster together and is defined as

$$C=\frac{1}{N}\sum_{i}C_{i},$$ (3)

where *N* is the number of nodes in the network, and *C*_*i*_ is the clustering coefficient of node *i* which is given by:

$$C_{i}=\frac{E_{i}}{k_{i}(k_{i}-1)/2},$$ (4)

where *k*_*i*_ is the degree of node *i*, and *E*_*i*_ is the number of the links among the neighbors of node *i*. Note that for a node to be the neighbor of node *i* implies that there is a link between the two. Denote the number of neighbors of node *i* to be *k*_*i*_, the largest number of links among *k*_*i*_ nodes is then equal to *k*_*i*_(*k*_*i*_− 1)/2, and *C*_*i*_ is between 0 and 1. In general, the larger the clustering coefficient of one node is, the more the nodes in the network will cluster around this node and renders it to be more important.

**Fig 5:**
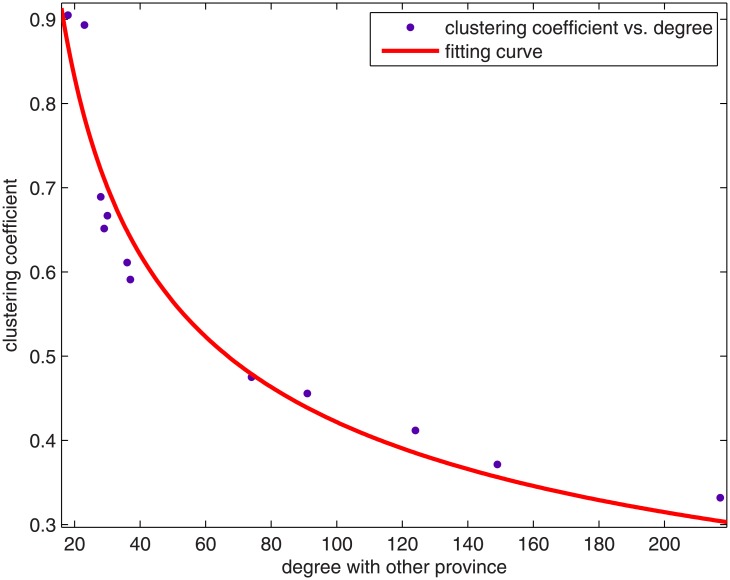
Clustering coefficient of a province/district as a function of its out-degree. Blue point: Clustering coefficient vs. degree; Red line: fitting curve.

Similar to Section 3.2, we study the clustering coefficient of a province/district as a whole, since we want to relate this to the economic activities of the province/district. We therefore build a new network for calculating each province’s clustering coefficient based on our initial VC network. If syndication exists between two provinces, they will be connected by a link. In some cases, there are many links between two provinces or districts. We can view this as the economic activities between the provinces—the more links they have, the more the economic activities between the two. We thus have a new network with each node corresponding to a province or district. We can calculate the clustering coefficient of each node of this new network and the result is given in Table 2. Notice that if a region has no links with any other regions or only connects to one region, its clustering coefficient will be set to 1. We can further plot the clustering coefficient of each province/district against their “out-degree”. The “out-degree” here means that it is the number of links that the province/district connects with other provinces and districts. Fig 5 shows the relation between clustering coefficient and the out-degree of a province/district with out-degree larger than 18. One can fit the empirical data set with an approximate power law function

$$y=2.94x^{-0.42},$$ (5)

where *y* denotes the clustering coefficient of the province/district and *x* denotes its out-degree.

**Table 2:** Clustering coefficient of each province/district in the VC network of China.

Province	Clustering coefficient	Province	Clustering coefficient
Anhui	0.73	Nei Monggol	1
Beijing	0.41	Ningxia	1
Fujian	0.60	Shandong	0.59
Guangdong	0.33	Shanxi	1
Guangxi	0.87	Shanxi(Xian)	0.93
Hainan	0.87	Shanghai	0.37
Hebei	0	Sichuan	0.89
Henan	0.65	Tianjin	0.68
Heilongjiang	1	Xizang	1
Hubei	0.61	Hong Kong	0.90
Hunan	0.67	Xinjiang	1
Jilin	1	Yunnan	0
Jiangsu	0.46	Zhejiang	0.47
Jiangxi	1	Chongqing	0.80
Liaoning	0.83		

### Robustness

For a stable network, there should be no abrupt change of the number of its isolated points and the mean shortest paths when its nodes are removed. We here study the robustness of the network by using two methods to remove the nodes and observe the increase of the number of the isolated points:

Method 1: Remove the nodes randomly from 0% to 30% (average the results after running 1000 times).
Method 2: Remove the nodes selectively from 0% to 30%. We sort the nodes according to their degrees, and remove the nodes starting from nodes with the highest degree.

**Fig 6:**
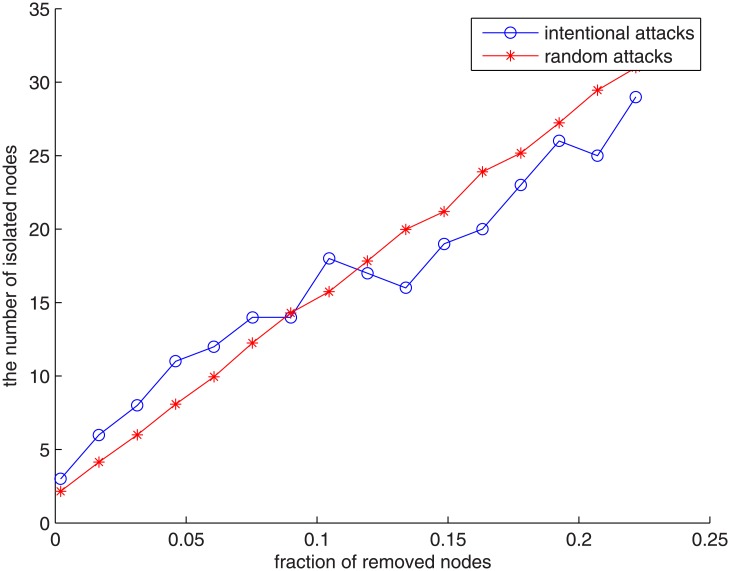
Number of isolated nodes vs. fraction of removed nodes in the VC network. The red curve refers to random attacks (Method 1) while the blue curve is for intentional attacks (Method 2). Circle point: intentional attacks; Star point: random attacks.

Method 1 can be viewed as random attacks while Method 2 refers to intentional attacks. Fig 6 shows the number of isolated points vs. the fraction of removed nodes in the VC network. It is easy to see that the results from these two methods show similar behavior. In both cases, the number of isolated points is roughly proportional to the fraction of removed nodes in the VC network. The network is therefore robust under either random attacks or intentional attacks. This reveals the fact that there are no dominant venture capital firms in China which act as hubs in the VC network.

### Topological Properties of the Venture Capital Network

In this section, we analyze the topological properties of the VC network of China. We will here investigate whether the VC network has the small-world, scale-free or self-similarity property.

A small-world network is one which has small mean shortest path and large clustering coefficient. Many natural networks display small-world behavior since one can reach a node from a designated node in a small number of steps. The mean shortest path and mean clustering coefficient of the Chinese VC network are 4.83 and 0.86 respectively, which is smaller in shortest path length and larger in clustering coefficient than regular networks with the same number of nodes and edges. The result suggests that the network has small-world behavior. Mathematically, the small-world property can be expressed by a slow (logarithmic) increase of the average length of the shortest paths $\bar{l}$, with respect to the total number of nodes, *N* in the network, i.e. $\bar{l}\sim \text{ln}N$, or

**Fig 7:**
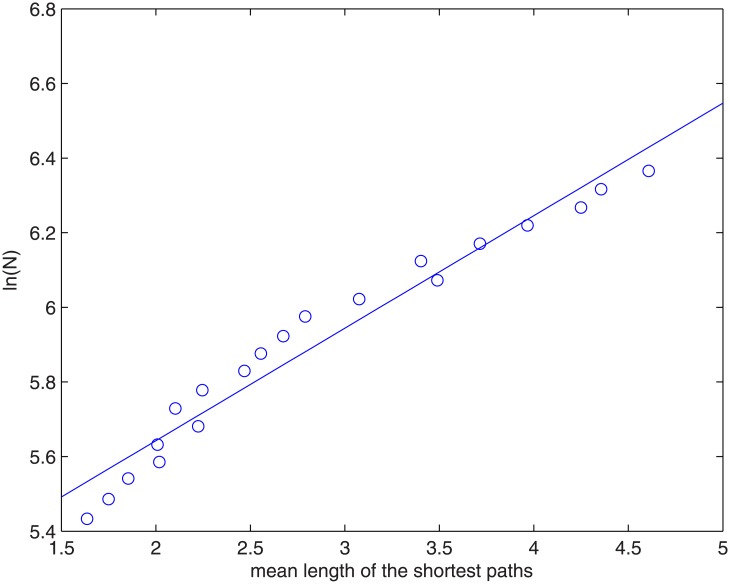
A plot of ln(*N*) vs. $\bar{l}$ for the Chinese VC network. The open circles are the empirical data points and the blue line is its linear fit.

N~el¯l0, (6)

where *l*_0_ is a characteristic length scale. We rewrite Eq (6) as

$$\text{ln}N=\text{ln}C+\frac{\bar{l}}{l_{0}},$$ (7)

where *C* is a constant. Fig 7 is a plot of In(*N*) vs. $\bar{l}$ for the VC network. One can see that it follows approximately a linear function. A linear fit gives

$$\text{ln}N=5.04+0.30\bar{l}$$ (8)

When the probability distribution of the link per node, *p*(*k*) (also known as the degree distribution) follows a power-law with an exponent of degree, *r*, i.e. *p*(*k*)~*k* ^−*r*^, the network is said to be scale-free. The most notable characteristic of a scale-free network is the attributions of vertices with a degree that greatly exceeds the average. The highest-degree nodes are often called hubs, and are thought to serve specific purposes in their networks, although this depends greatly on their locations.

Fig 4 shows a plot of the cumulative degree distribution of the VC network in China. The empirical data points do not seem to fall on a straight line so we use exponential functions to fit the empirical dataset instead of a power law fit. The result gives

$$p=\{\begin{array}{c} e^{-0.39k+5.50},k<7\\ e^{-0.27k-1.41},k\geq 7 \end{array}$$ (9)

where *p* is the cumulative probability density function of the degree distribution. The scale-free property and the robustness of a network are indeed strongly correlated. If some of the hubs are removed, there will be a drastic increase of the number of the isolated points and the mean shortest path of the network. Since no such drastic change appears in Fig 4, the VC network is therefore not scale-free. This is in agreement with earlier findings by other groups (Kogut et al., 2007; Mas et al., 2008) on the study of VC networks which states that the links are not purely determined by preferential attachment, and differs from the structure of numerous well known social networks.

We should mention here that we have also studied the self-similarity property of the VC network by using the method introduced in Zhou et al. (2007). The relatively small Chinese VC network is unable to exhibit any self-similarity behavior. One would hope that when there are more venture capital firms in China, one would be able to investigate its self-similarity property in the future.

## The Relationship with Regional Economy of the VC Network

### Hypotheses

Venture capital is an important part of a social economic system. The growth of venture capital investment of an area makes a great contribution to the local economy. On the other hand, the development of the economy of an area also facilitates the pace of venture capital investment. In the previous section, we calculate several parameters related to the statistical properties of the Chinese VC network. We are interested in exploring the relationship between the statistical properties of the VC network and the economic indicators of an economic system. We will employ multivariate linear regression models to probe into this issue in this section.

#### Economic Aggregate

Venture capital not only invests money in a new venture, but also provides some value-added service to it. Many new ventures, which receive fund and services from venture capital, such as Microsoft, Apple, Yahoo, and Amazon, grow up faster and steadier than those without investment from venture capital. Since venture capital can add value to the enterprises invested, it can therefore make contribution to the development of regional economy, especially to the increase of economic aggregate of a region. The leading index which indicates the economic aggregate is undoubtedly GDP (gross domestic product). In general, the total GDP comprises four components, namely investment, consumption, net export and government expenditures. The first three parts comprise the main body of GDP, and are usually called as “three-in-hand” of GDP. Hence, we put forward the following hypotheses:

H1: The economic aggregate of a region is positively correlated with the degree and the clustering coefficient of the VC sub-network of the region.

#### Upgrade of industrial structure

Traditional economists divide the national economy into three industries, namely primary, secondary and tertiary industries. The industry whose subjects of labor come directly from nature is called primary industry, e.g., agriculture. Secondary industry is what reprocesses primary products, such as the manufacturing industry, and tertiary industry provides services for the process of production and consumption, e.g. the IT industry. During the development of a national economy, its main body will convert from primary to secondary and then to tertiary industry. Industrial structure of a national economy is generally referred as the composition of industries and the connection and proportional relation of industries. Upgrade of industrial structure is a primary target of the government of a nation. There are many indices to measure the effects of the upgrade of industrial structure, e.g. the total amount of tertiary industry, the turnover of technological market and the energy consumption per ten thousand yuan of GDP (one yuan is about 0.16 US dollar in the month of July, 2014), etc.

As an important investment mechanism of new ventures which are usually the testing field of high technology, it is hoped that venture capital can shoulder heavy responsibility in upgrading the industrial structure. We will test if this is the case, using data from Chinese regional economies. We put forward the following hypothesis.

H2: The upgrade of industrial structure of a region is positively correlated with the degree and the clustering coefficient of the VC sub-network of the region.

#### Employment

One of the primary targets of a government is to increase employment level. From the viewpoint of society and government, the criterion of evaluating the effect of an economic entity or an economic action is to judge if the economic entity or the economic action can help to increase the employment level. As an important investment mechanism, venture capital provides many growth opportunities to new ventures. Many new ventures emerge and blossom from the support of venture capital. This undoubtedly contributes a lot to the increase of employment of a society. This leads to the following hypothesis:

H3: The employment rate of a region is positively correlated with the degree and the clustering coefficient of the VC subnet of the region.

#### Remuneration

Like employment, the people’s livelihood is not only a prior consideration of a government, but also an important measurement criterion for the performance of a government. The remuneration of the resident of a region is an important index to judge if a government achieves the aim of improving people’s livelihood. It will be a measure of the role of venture capital in promoting the remuneration of the resident of a region in its economic development. We put forward the following hypothesis.

H4: The remuneration of the resident of a region is positively correlated with the degree and the clustering coefficient of the VC subnet of the region.

### Description of data and choice of Variables

#### Choice of variables

We now set up linear univariate regression models to test the hypotheses above. We will investigate the relation between the characteristics of VC network of China, mainly degrees and clustering coefficients, and regional economic indices. To study the effect of the VC network on the regional economies, we use the degrees and clustering coefficients of regional sub-networks as the independent variables of the multivariate linear regression models. The degrees and clustering coefficients of each province/district are shown in Tables 1 and 2.

For the dependent variables, we choose 12 economic indices to express the four aspects of regional economy, namely economic aggregate, upgrade of industrial structure, employment and remuneration. In the aspect of economic aggregate, we choose total GDP to represent the aggregate economic output, and choose the gross capital formation, the consumption level and net export to represent investment, consumption and net export. Upgrade of industrial structure is represented by the value-added of tertiary industry, R&D funds of industrial enterprises above designated size and the number of patent applications accepted. For employment, we adopt three variables in the study, namely the number of industrial enterprises, the number of private enterprises, and the employment in urban units. Finally, we select two variables to represent remuneration—the remuneration for workers and the urban per capita disposable income. The economic data are taken from 2011 and 2012.

Detailed information of the 12 economic indices of each province/district in the VC network is presented in S1 Appendix. From Table A1 within SI Appendix, one can see that the economic indices of the eastern developed provinces/districts, namely Beijing, Shanghai, Guangdong, Jiangsu and Zhejiang, are larger values than those of the western underdeveloped provinces/districts, such as Yunnan, Ningxia, Xinjiang and Shanxi (Taiyuan), etc. We will check if there is a significant relation between each economic index and the degree or clustering coefficient of each province/district. We will perform a multivariate linear regression analysis to explore this issue.

To improve accuracy, we here add three control variables, namely the ratio consumption level between town and country (CV1), gas emission of sulfur dioxide (CV2) and the rate of population growth (CV3). These control variables come from three different aspects. CV1 is about people's livelihood, the CV2 index represents environment, and the CV3 refers to population. All these three indices are not correlated with economic indices directly, so they can be used to some extent as instrument variables to overcome endogeneity.

#### Description of data

As depicted above, there are three kinds of data, namely economic indices, non-economic indices (control variables), and the network’s characteristics indices in our multivariate linear regression models. We here choose 12 economic indices as dependent variables, 3 non-economic indices as control variables, and 2 network’s characteristics indices as independent variables. We list the basic statistics values of these data in Table A2 of S1 Appendix [44]. 12 economic indices are from 4 aspects, namely economic aggregate, upgrade of industrial structure, employment and remuneration. In order to eliminate multi-collinearity and to facilitate the analysis, we use main components analysis method to extract two main components from the four indices of economic aggregate. In the same way, we extract one main component from the three indices of upgrade of industrial structure and the three indices of employment respectively. Since there are only two indices in remuneration, there is no need to run main components analysis for these two indices.

### Multivariate linear regression results and analysis

We here perform an analysis of the effect of the statistical properties of the Chinese VC network on the chosen economic indices from four aspects which are important in a regional economy, namely economic aggregate, upgrade of industrial structure, employment and remuneration. The 12 economic indices, the degree and the clustering coefficient are first normalized in the usual manner. We get the standard score *z* by the following process

$$z=\frac{X-u}{\sigma }$$

where *X* is any one of the economic indices, degree or clustering coefficient from raw data, *u* is its mean and σ is its standard deviation.

#### Economic aggregate

Generally speaking, GDP is the most important index to measure the economic performance of a nation/region. It mainly comprises investment, consumption and net export. To investigate the relation between the characteristics of VC network and the economic aggregate of a region, we make linear regression models (1) and (2). The result is presented in Table 3. The independent variables of Models (1) and (2) are the clustering coefficient and degree of a region, and the dependent variables are the two main components of the total GDP, total capital formation, consumption level and total trade respectively. Besides, we introduce three control variables, namely the ratio of the consumption level between town and country, gas emission of sulfur dioxide, and the rate of population growth.

**Table 3:** Result of regression of characteristics of VC network and the economic aggregate of a region in 2011.

		component1/Model(1)	component2/Model(2)	Component1/Model(1)	Component2/Model(2)
independent variable	degree	0.89/(0.00***)	-0.91/(0.00***)		
	clustering coefficient			-0.48/(0.015*)	0.47/(0.017*)
control value	The ratio of consumption level between town and city	-0.26	0.25	-0.01	-0.09
	Gas emission of sulfur dioxide	0.25	-0.092	0	0.13
	The rate of population growth	-0.89	-0.034	-0.14	0.14
	Multiple R-squared	0.87	0.89	0.28	0.28
	P-value	0.00	0.00	0.10	0.10

Significant codes: 0 ‘***’ 0.001, 0.01 ‘*’ 0.05.

From Tables 3 and 4, we see that, in models (1) to (2), the t-values of the independent variables are all significant, not only in the same year (2011), but also in the following year (2012). Thus, we demonstrate that the hypothesis H1 is in agreement with the empirical data. In other words, the characteristics of Chinese VC network are significantly correlated with the regional economic aggregate of China.

**Table 4:** Result of regression of characteristics of VC network and the economic aggregate of a region in 2012.

		Component 1/Model(1)	component 2/Model(2)	Component 1/Model(1)	Component 2/Model(2)
independent variable	degree	0.93/(0.00***)	-0.94/(0.00***)		
	clustering coefficient			-0.48/(0.02*)	0.47/(0.02*)
control value	The ratio of consumption level between town and city	-0.26	0.25	-0.073	0.059
	Gas emission of sulfur dioxide	0.22	-0.083	-0.19	0.14
	The rate of population growth	0.062	-0.055	-0.14	0.15
	Multiple R-squared	0.87	0.90	0.27	0.27
	P-value	0.00	0.00	0.11	0.12

Significant codes: 0 ‘***’ 0.001, 0.01 ‘*’ 0.05.

#### Upgrade of industrial structure

Upgrade of industrial structure is a major indication of the economic development of a nation. There are many measures to evaluate the effect of the upgrade of industrial structure. We here choose three indices to represent the effect of upgrade of industrial structure, namely the total amount of tertiary industry, R&D funds of industrial enterprises above designated size and the number of patent applications accepted. We use linear regression model (3) to test the relation between the characteristics of the VC network and the upgrade of industrial structure of a region. The result is presented in Table 4. The independent variables of models (3) are also the clustering coefficient and degree of a region, and the main component of the value-added of tertiary industry, R&D funds of industrial enterprises above designated size and the number of patent applications accepted is the dependent variable. The three control variables are the same as models (1) and (2).

Table 5 shows that the t-values of the independent variables are significant both in 2011 and the following year (2012). Hypothesis H2 therefore agrees with the empirical data. In other words, the characteristics of Chinese VC network are significantly correlated with the upgrade of industrial structure of a region in China.

**Table 5:** Result of regression of the characteristics of Chinese VC network and the upgrade of industrial structure of a region.

		Component/2011/model(3)	Component/2012/model(3)	Component/2011/model(3)	Component/2012/model(3)
independent variable	degree	0.57/(0.00***)	0.58/(0.00***)		
	clustering coefficient			-0.36/(0.06)	0.33/(0.08.)
control value	The ratio of consumption level between town and city	-0.43	-0.46	-0.31	0.34
	Gas emission of sulfur dioxide	0.52	0.36	0.21	0.21
	The rate of population growth	-0.31	0.58	-0.11	0.079
	Multiple R-squared	0.65	0.52	0.31	0.30
	P-value	0.00	0.002	0.07	0.08

Significant codes: 0 ‘***’ 0.001, 0.05 ‘.’ 0.1.

#### Employment

Improving the employment rate is always a primary duty of a government. Venture capital can add value to new ventures by raising their survival rates and enlarging their scales, and further create job opportunities. The development of VC can intuitively help to improve the employment rate of a nation/region. We here use the linear regression model (4) to test the relation between the characteristics of VC network and the employment of a region. The result is presented in Table 6. The independent variables of models (4) are the clustering coefficient and degree of a region and the dependent variable is the main component of the number of industrial enterprises, the number of private enterprises and the employment in urban units. We also use three control variables which are the same as in models (1) and (2).

**Table 6:** Result of regression of the characteristics of VC network and the employment of a region.

		Component/2011/model(4)	Component/2012/model(4)	Component/2011/model(4)	Component/2012/model(4)
independent variable	degree	0.54/(0.003***)	0.49/(0.003***)		
	clustering coefficient			-0.36/(0.04*)	0.33/(0.05.)
control value	The ratio of consumption level between town and city	-0.34	-0.39	-0.26	-0.28
	Gas emission of sulfur dioxide	0.74	0.62	0.42	0.49
	The rate of population growth	-0.33	0.10	-0.039	-0.012
	Multiple R-squared	0.61	0.49	0.39	0.42
	P-value	0.00	0.001	0.02	0.01

Significant codes: 0 ‘***’ 0.001, 0.01 ‘*’ 0.05, 0.05 ‘.’ 0.1.

Table 6 indicates that the clustering coefficients and degrees of the Chinese regional VC network are significantly correlated with the main component of the number of industrial enterprises, the number of private enterprises and the employment in urban units of a region in 2011 as well as 2012. The empirical data are in agreement with hypothesis H3. In other words, the characteristics of Chinese VC network are significantly correlated with employment level of a region in China.

#### Remuneration

Remuneration of a nation is an important indicator of the living standard of the residents of a nation. Therefore, enhancing the remuneration of a region will be an evaluation criterion of the effect of an economic unit or an economic action. As a distinctive financing system of new ventures, venture capital should play a role in increasing the remuneration of a region. We use two linear regression models (5) and (6) to study the relation between the characteristics of VC network and the remuneration of a region. The result is presented in Tables 7 and 8. The independent variables of models (5) and (6) are the clustering coefficient and degree of a region, and the dependent variables are the remuneration for workers and the urban per capita disposable income respectively. Three control variables which are the same as in models (1) and (2) are included as well.

**Table 7:** Result of regression of characteristics of the Chinese VC network and the remuneration of a region in 2011.

		remuneration for workers/2011/model(5)	the urban per capita disposable income/2011/model(6)	remuneration for workers/2011/model(5)	the urban per capita disposable income/2011/model(6)
independent variable	degree	0.62/(0.00***)	0.61/(0.00***)		
	clustering coefficient			-0.47/(0.01**)	-0.48/(0.00**)
control value	The ratio of consumption level between town and city	-0.23	-0.47	-0.11	-0.28
	Gas emission of sulfur dioxide	0.72	-0.66	0.46	-0.25
	The rate of population growth	-0.19	0.00	-0.082	-0.23
	Multiple R-squared	0.73	0.62	0.49	0.51
	P-value	0.00	0.00	0.004	0.003

Significant codes: 0 ‘***’ 0.001 ‘**’ 0.01.

**Table 8:** Result of regression of characteristics of the Chinese VC network and the remuneration of a region in 2012.

		remuneration for workers/2012/model(5)	the urban per capita disposable income/2012/model(6)	remuneration for workers/2012/model(5)	the urban per capita disposable income/2012/model(6)
independent variable	degree	0.68/(0.00***)	0.61/(0.00***)		
	clustering coefficient			-0.47/(0.01**)	-0.48/(0.00**)
control value	The ratio of consumption level between town and city	-0.27	-0.43	-0.11	-0.28
	Gas emission of sulfur dioxide	0.64	-0.07	-0.46	-0.25
	The rate of population growth	0.07	-0.08	-0.088	-0.23
	Multiple R-squared	0.71	0.63	0.49	0.51
	P-value	0.00	0.00	0.004	0.003

Significant codes: 0 ‘***’ 0.001 ‘**’ 0.01.

Tables 7 and 8 suggest that the clustering coefficients and degrees of the Chinese regional VC network are significantly correlated with the remuneration for workers and the urban per capita disposable income in 2011 as well as in 2012. Hence, hypothesis H4 is supported by empirical data. In other words, the characteristics of Chinese VC network are significantly correlated with remuneration level of a region in China.

## Conclusion

In this paper, we studied the network of venture capital firms in China using the data from Chinese GEM and SME. We computed and analyzed the basic statistical properties of the network, namely the network degrees, mean lengths of the shortest paths, clustering coefficients and robustness. We further studied the topological properties of the Chinese VC network and found that it has small-world structure. From the study of its statistical and topological properties, we found that there are no dominant venture capital firms in China which act as hubs in the VC network. Furthermore, the Chinese VC network possesses a small-world structure but is not scale-free. This is in agreement with earlier findings by other groups (Kogut et al., 2007; Mas et al., 2008) on the study of VC networks which states that the links are not purely determined by preferential attachment.

In order to understand the network structure from the point of view of the economic activities of a region, we constructed four multivariate linear regression models to study the relation between network parameters and major regional economic indices, which we categorized into four aspects, i.e. economic aggregate, upgrade of industrial structure, employment and remuneration. From the result of regression, we find that, economic aggregate (including the total GDP, investment, consumption and net export), upgrade of industrial structure, employment and remuneration of a region are all positively correlated with the degree and the clustering coefficient of the VC sub-network of the region, which suggests that the development of the VC industry has substantial effects on regional economy in China.

From the above results, we can conclude that similar to the US, the VC industry in China, though still small, plays an important role in the development of a region. VC is a booster of the development of high technology industry. The involvement of VC promotes many high technology ventures to grow. These high technology ventures are the props of regional economy. Hence, it is easy to understand that VC industry has a significant positive relationship with the regional economy. Although in China, the role of VC industry in regional economy is still restricted, the prospect of the development of VC industry in China is bright. The government should give more attention to the development of VC industry and launch more favorable policies to propel the growth of VC industry.

China continues to be a rapidly changing environment and its venture capital will continue to grow. The standards and professionalism of the VC industry in the country are also improving. It would also be interesting to study the effect of other financial networks on the economy of a region and compare the results. One would then check if there are correlations among various networks and would therefore extract useful information from these studies.

## Supporting Information

S1 Appendix *Table A1*, Economic indices of each province/district for 2011 and 2012. *Table A2*, The basic statistics data of the variables. *Table A3*, The main component of the economic indices of each aspect. *Table A4*, The three control variables of each province/district.
(DOC)
