# Supplementary material for: Characteristics of Venture Capital Network and Its Correlation with Regional Economy: Evidence from China
Source: PLoS One. 2015 Sep 4;10(9):e0137172. doi: 10.1371/journal.pone.0137172 (PMC4560442; doi:10.1371/journal.pone.0137172)
Supplement: S1 Appendix — Table A1, Economic indices of each province/district for 2011 and 2012. Table A2, The basic statistics data of the variables. Table A3, The main component of the economic indices of each aspect. Table A4, The three control variables of each province/district. (DOC) [file pone.0137172.s001.doc]

**Appendix**

Table A1. Economic indices of each province/district for 2011 and 2012.

|  | Total GDP (hundred million yuan) | | Gross capital formation (hundred million yuan) | | The consumption level | | Total volume of foreign trade (Million US dollars) | | The value-added of tertiary industry  (hundred million yuan) | | R&D funds of industrial enterprises above designated size (hundred million yuan) | |
| --- | --- | --- | --- | --- | --- | --- | --- | --- | --- | --- | --- | --- |
|  | 2011 | 2012 | 2011 | 2012 | 2011 | 2012 | 2011 | 2012 | 2011 | 2012 | 2011 | 2012 |
| Anhui | 15301 | 17212 | 7725 | 8856 | 10055 | 10978 | 31309 | 39285 | 4976 | 5628 | 163 | 209 |
| Beijing | 16252 | 17879 | 6684 | 7410 | 27760 | 30350 | 389556 | 408107 | 12363 | 13670 | 165 | 197 |
| Fujian | 17560 | 19702 | 10075 | 11305 | 14958 | 16144 | 143522 | 155938 | 6879 | 7737 | 194 | 238 |
| Guangdong | 53210 | 57068 | 21004 | 22872 | 19578 | 21823 | 913467 | 984020 | 24098 | 26520 | 899 | 1078 |
| Guangxi | 11721 | 13035 | 9989 | 11068 | 9181 | 10519 | 23356 | 29484 | 3998 | 4615 | 59 | 70 |
| Hainan | 2523 | 2856 | 1497 | 2010 | 9238 | 10634 | 12756 | 14322 | 1149 | 1340 | 6 | 8 |
| Hebei | 24516 | 26575 | 13890 | 15245 | 9551 | 10749 | 53601 | 50563 | 8483 | 9385 | 159 | 198 |
| Henan | 26931 | 29599 | 19167 | 22060 | 9171 | 10380 | 32623 | 51739 | 7992 | 9158 | 214 | 249 |
| Helongjiang | 12582 | 13692 | 6888 | 8144 | 10634 | 11601 | 38523 | 37590 | 4550 | 5540 | 84 | 91 |
| Hubei | 19632 | 22250 | 11027 | 12555 | 10873 | 12283 | 33587 | 31964 | 7247 | 8209 | 211 | 263 |
| Hunan | 19670 | 22154 | 10913 | 12489 | 10547 | 11740 | 18944 | 21949 | 7540 | 8644 | 182 | 229 |
| Jilin | 10569 | 11939 | 8207 | 9136 | 10811 | 12276 | 22061 | 24563 | 3680 | 4150 | 49 | 60 |
| Jiangsu | 49110 | 54058 | 25049 | 27258 | 17167 | 19452 | 539581 | 547961 | 20842 | 23518 | 900 | 1080 |
| Jiangxi | 11703 | 12949 | 5989 | 6514 | 9523 | 10573 | 31469 | 33414 | 3921 | 4486 | 77 | 93 |
| Liaoning | 22227 | 24846 | 13930 | 15492 | 15635 | 17999 | 96036 | 104090 | 8159 | 9460 | 275 | 289 |
| Nei Monggol | 14360 | 15881 | 11015 | 13442 | 13264 | 15196 | 11931 | 11259 | 5016 | 5631 | 70 | 86 |
| Ningxia | 2102 | 2341 | 1755 | 2087 | 10492 | 12120 | 2286 | 2217 | 862 | 983 | 12 | 14 |
| Shandong | 45362 | 50013 | 24944 | 27552 | 13565 | 15095 | 235887 | 245544 | 17371 | 19996 | 743 | 906 |
| Shanxi  (Taiyuan) | 11238 | 12113 | 7251 | 8224 | 9746 | 10829 | 14743 | 15043 | 3961 | 4683 | 90 | 107 |
| Shanxi  （Xi’an） | 12512 | 14454 | 8487 | 9915 | 10054 | 11852 | 14647 | 14799 | 4356 | 5010 | 97 | 119 |
| Shanghai | 19196 | 20182 | 7729 | 7675 | 35439 | 36893 | 437549 | 436587 | 11143 | 12199 | 344 | 372 |
| Sichuan | 21027 | 23873 | 11068 | 12496 | 9903 | 11280 | 47724 | 59144 | 7014 | 8242 | 104 | 142 |
| Tianjin | 11307 | 12894 | 8594 | 9848 | 20624 | 22984 | 103376 | 115634 | 5219 | 6058 | 211 | 256 |
| Xinjiang | 6610 | 7505 | 4162 | 5792 | 8895 | 10675 | 22820 | 25170 | 2245 | 2703 | 22 | 27 |
| Yunnan | 8893 | 10309 | 7139 | 8576 | 8278 | 9782 | 16029 | 21014 | 3702 | 4236 | 30 | 38 |
| Zhejiang | 32319 | 34665 | 14744 | 15461 | 21346 | 22845 | 309378 | 312401 | 14180 | 15681 | 480 | 589 |
| Chongqing | 10011 | 11410 | 5758 | 6341 | 11832 | 13655 | 29208 | 53204 | 3624 | 4494 | 94 | 117 |

Table A1. (continued) Economic indices of each province/district for 2011 and 2012.

|  | The number of patent applications accepted | | The number of industrial enterprises | | The number of private enterprises (ten thousands) | | the employment in urban units (ten thousands persons) | | Remuneration for workers (hundred million yuan) | | the urban per capita disposable income (yuan) | |
| --- | --- | --- | --- | --- | --- | --- | --- | --- | --- | --- | --- | --- |
|  | 2011 | 2012 | 2011 | 2012 | 2011 | 2012 | 2011 | 2012 | 2011 | 2012 | 2011 | 2012 |
| Anhui | 48556 | 74888 | 12432 | 14514 | 26.30 | 30.43 | 412 | 437 | 7435 | 8445 | 18606 | 21024 |
| Beijing | 77955 | 92305 | 3746 | 3692 | 54.40 | 60.04 | 686 | 717 | 7992 | 9103 | 32903 | 36469 |
| Fujian | 32325 | 42773 | 14116 | 15333 | 30.70 | 34.74 | 596 | 638 | 8742 | 9979 | 24907 | 28055 |
| Guangdong | 196272 | 229514 | 38305 | 37790 | 110.80 | 125.62 | 1238 | 1304 | 24288 | 27240 | 26898 | 30227 |
| Guangxi | 8106 | 13610 | 5046 | 5239 | 18.10 | 22.69 | 342 | 358 | 6806 | 7183 | 18854 | 21243 |
| Hainan | 1489 | 1824 | 358 | 377 | 9.10 | 10.22 | 85 | 90 | 1273 | 1447 | 18369 | 20918 |
| Hebei | 17595 | 23241 | 11570 | 12360 | 30.40 | 33.9 | 555 | 620 | 12497 | 13657 | 18292 | 20543 |
| Henan | 34076 | 43442 | 18328 | 19237 | 35.00 | 39.36 | 839 | 881 | 13439 | 14835 | 18195 | 20443 |
| Helongjiang | 23432 | 30610 | 3377 | 3911 | 17.40 | 19.78 | 466 | 471 | 4616 | 5418 | 15696 | 17760 |
| Hubei | 42510 | 51316 | 10633 | 12441 | 30.80 | 34.58 | 586 | 598 | 9433 | 10814 | 18374 | 20840 |
| Hunan | 29516 | 35709 | 12477 | 12785 | 21.90 | 24.89 | 551 | 567 | 9802 | 10988 | 18844 | 21319 |
| Jilin | 8196 | 9171 | 5158 | 5286 | 14.20 | 16.04 | 278 | 285 | 4086 | 4589 | 17797 | 20208 |
| Jiangsu | 348381 | 472656 | 43368 | 45859 | 119.80 | 131.29 | 811 | 831 | 20523 | 22868 | 26341 | 29677 |
| Jiangxi | 9673 | 12458 | 6481 | 7217 | 19.40 | 22.59 | 344 | 386 | 5144 | 5529 | 17495 | 19860 |
| Liaoning | 37102 | 41152 | 16914 | 17347 | 33.70 | 37.4 | 580 | 599 | 10269 | 11560 | 20467 | 23223 |
| Nei Monggol | 3841 | 4732 | 4175 | 4244 | 13.30 | 14.66 | 262 | 271 | 6240 | 6961 | 20408 | 23150 |
| Ningxia | 1079 | 1985 | 764 | 865 | 4.20 | 4.58 | 61 | 67 | 1062 | 1151 | 17579 | 19831 |
| Shandong | 109599 | 128614 | 35813 | 37625 | 59.80 | 66.05 | 1050 | 11107 | 17444 | 19235 | 22792 | 25755 |
| Shanxi  (Taiyuan) | 12769 | 16786 | 3675 | 3905 | 18.30 | 20.28 | 410 | 436 | 4675 | 5319 | 18124 | 20412 |
| Shanxi  （Xi’an） | 32227 | 43608 | 3684 | 4284 | 21.60 | 23.79 | 394 | 411 | 4912 | 5567 | 18245 | 20734 |
| Shanghai | 80215 | 82682 | 9962 | 9772 | 77.70 | 84.71 | 497 | 556 | 7710 | 8389 | 36231 | 40188 |
| Sichuan | 49734 | 66312 | 12085 | 12719 | 40.50 | 44.82 | 614 | 641 | 9382 | 10538 | 17899 | 20307 |
| Tianjin | 38489 | 41009 | 5013 | 5342 | 15.90 | 17.32 | 268 | 289 | 4378 | 5040 | 26921 | 29626 |
| Xinjiang | 4736 | 7044 | 1738 | 1959 | 10.70 | 11.67 | 279 | 289 | 3345 | 3979 | 15514 | 17921 |
| Yunnan | 7150 | 9260 | 2773 | 3211 | 17.90 | 20.29 | 350 | 393 | 4271 | 5214 | 18576 | 21075 |
| Zhejiang | 177066 | 249373 | 34698 | 36496 | 71.90 | 77.53 | 996 | 1070 | 13186 | 14584 | 30971 | 34550 |
| Chongqing | 32039 | 38924 | 4778 | 4985 | 22.80 | 29.22 | 337 | 353 | 4930 | 5679 | 20250 | 22968 |

Table A2. The basic statistics data of the variables.

|  | Total GDP (hundred million yuan) | | | Gross capital formation (hundred million yuan) | | | | The consumption level | | | |
| --- | --- | --- | --- | --- | --- | --- | --- | --- | --- | --- | --- |
| year | 2010.00 | 2011.00 | | 2010.00 | 2011.00 | | | 2010.00 | | | 2011.00 |
| maximum | 53210.00 | 57068.00 | | 25049.00 | 27552.00 | | | 35439.00 | | | 36893.00 |
| minimum | 2102.00 | 2341.00 | | 1497.00 | 2010.00 | | | 8278.00 | | | 9782.00 |
| mean | 18831.26 | 20794.59 | | 10543.70 | 11845.30 | | | 13634.07 | | | 15211.37 |
| variance | 168293027.58 | 196989942.64 | | 37183304.06 | 43798970.14 | | | 41643892.38 | | | 44778017.93 |
|  | Total volume of foreign trade (Million US dollars) | | | The value-added of tertiary industry  (hundred million yuan) | | | | R&D funds of industrial enterprises above designated size (hundred million yuan) | | | |
| year | 2010.00 | | 2011.00 | 2010.00 | 2011.00 | | | 2010.00 | | | 2011.00 |
| maximum | 913467.00 | | 984020.00 | 24098.00 | 26520.00 | | | 900.00 | | | 1080.00 |
| minimum | 2286.00 | | 2217.00 | 862.00 | 983.00 | | | 6.00 | | | 8.00 |
| mean | 134295.15 | | 142481.67 | 7576.67 | 8591.70 | | | 219.78 | | | 263.89 |
| variance | 45701910648.28 | | 50171756653.31 | 33255225.08 | 40798066.60 | | | 62985.18 | | | 90774.79 |
|  | The number of patent applications accepted | | | The number of industrial enterprises | | | | The number of private enterprises (ten thousands) | | | |
| year | 2010.00 | | 2011.00 | 2010.00 | | 2011.00 | | 2010.00 | | 2011.00 | |
| maximum | 348381.00 | | 472656.00 | 43368.00 | | 45859.00 | | 119.80 | | 131.29 | |
| minimum | 1079.00 | | 1824.00 | 358.00 | | 377.00 | | 4.20 | | 4.58 | |
| mean | 54226.96 | | 69074.00 | 11906.19 | | 12547.96 | | 35.06 | | 39.20 | |
| variance | 5859651360.65 | | 10312061020.77 | 148239789.00 | | 158807314.50 | | 871.77 | | 1054.70 | |
|  | the employment in urban units (ten thousands persons) | | | Remuneration for workers (hundred million yuan) | | | | the urban per capita disposable income (yuan) | | | |
| year | 2010.00 | | 2011.00 | 2010.00 | 2011.00 | | | 2010.00 | | | 2011.00 |
| maximum | 1238.00 | | 11107.00 | 24288.00 | 27240.00 | | | 36231.00 | | | 40188.00 |
| minimum | 61.00 | | 67.00 | 1062.00 | 1151.00 | | | 15514.00 | | | 17760.00 |
| mean | 514.33 | | 913.52 | 8440.00 | 9455.96 | | | 21316.59 | | | 24012.07 |
| variance | 79832.23 | | 4225820.72 | 31063403.15 | 38112624.34 | | | 29086805.71 | | | 34294398.23 |
|  | Degree | | Clustering coefficient | the ratio of comsumption level between town and country | | | gas emission of sulfur dioxide | | The rate of population growth | | |
| year | 2010.00 | | 2010.00 | 2010.00 | | | 2010.00 | | 2010.00 | | |
| maximum | 385.00 | | 1.00 | 4.20 | | | 182.74 | | 13394.29 | | |
| minimum | 0.00 | | 0.00 | 2.10 | | | 3.26 | | 9.15 | | |
| mean | 49.44 | | 0.69 | 3.11 | | | 75.15 | | 2457.82 | | |
| variance | 7593.18 | | 0.09 | 0.32 | | | 1997.20 | | 10547957.51 | | |

Table A3. The main component of the economic indices of each aspect.

|  | 2011 | | | | 2012 | | | |
| --- | --- | --- | --- | --- | --- | --- | --- | --- |
|  | Model(1) | Model(2) | Model(3) | Model(4) | Model(1) | Model(2) | Model(3) | Model(4) |
| Anhui | 33175 | -8036 | 30513 | 7550 | 39530 | -10234 | 45411 | 9777 |
| Beijing | 233985 | -122811 | 51468 | 2624 | 246275 | -133808 | 59855 | 2787 |
| Fujian | 98317 | -41578 | 22432 | 8646 | 107352 | -46447 | 28639 | 10408 |
| Guangdong | 543848 | -245971 | 125789 | 23260 | 586266 | -277234 | 144826 | 25558 |
| Guangxi | 27656 | -5101 | 6949 | 3169 | 32762 | -7128 | 10362 | 3625 |
| Hainan | 12390 | -9147 | 1514 | 264 | 14170 | -10363 | 1808 | 295 |
| Hebei | 53464 | -8087 | 14990 | 7128 | 54162 | -7364 | 18617 | 8441 |
| Henan | 45983 | 1457 | 24082 | 11261 | 59834 | -3085 | 29847 | 13091 |
| Helongjiang | 35327 | -11755 | 15974 | 2260 | 36451 | -11611 | 20450 | 2798 |
| Hubei | 38837 | -6354 | 28430 | 6596 | 40767 | -5698 | 33721 | 8485 |
| Hunan | 30734 | -2021 | 21215 | 7653 | 35049 | -2406 | 25198 | 8692 |
| Jilin | 25973 | -7211 | 6810 | 3196 | 29107 | -8309 | 7596 | 3619 |
| Jiangsu | 339688 | -137950 | 210147 | 25992 | 349130 | -145920 | 279483 | 30669 |
| Jiangxi | 30148 | -9628 | 7804 | 4013 | 32575 | -10654 | 9654 | 4941 |
| Liaoning | 77378 | -25381 | 25930 | 10281 | 84911 | -28955 | 28762 | 11720 |
| Nei Monggol | 24918 | -3688 | 5128 | 2609 | 27340 | -3250 | 5982 | 2926 |
| Ningxia | 7040 | -7154 | 1119 | 486 | 7864 | -8039 | 1694 | 603 |
| Shandong | 171321 | -50751 | 72652 | 21660 | 181210 | -54776 | 84323 | 29755 |
| Shanxi | 21519 | -4601 | 9590 | 2403 | 23070 | -4829 | 12205 | 2779 |
| Shanxi(XIAN) | 22926 | -3811 | 20856 | 2401 | 25492 | -3977 | 27450 | 3020 |
| Shanghai | 264968 | -140799 | 52146 | 6178 | 265498 | -146296 | 53703 | 6738 |
| Sichuan | 46985 | -9154 | 32328 | 7470 | 56074 | -12480 | 42081 | 8693 |
| Tianjin | 74216 | -37223 | 24974 | 3106 | 83234 | -42514 | 26682 | 3659 |
| Xinjiang | 21407 | -9163 | 4000 | 1187 | 24646 | -10289 | 5541 | 1426 |
| Yunnan | 20307 | -4613 | 6219 | 1839 | 25085 | -6153 | 7683 | 2302 |
| Zhejiang | 201399 | -86427 | 108902 | 20981 | 205350 | -90952 | 149371 | 24572 |
| Chongqing | 28658 | -11384 | 20323 | 3011 | 43435 | -19341 | 24521 | 3459 |

Table A4. The three control variables of each province/district.

|  | the ratio of consumption level between town and country | gas emission of sulfur dioxide | The rate of population growth |
| --- | --- | --- | --- |
| Anhui | 3 | 52.95 | 1760.99 |
| Beijing | 2.1 | 9.79 | 348.61 |
| Fujian | 2.6 | 38.92 | 1304.14 |
| Guangdong | 4 | 84.77 | 809.94 |
| Guangxi | 3.9 | 52.1 | 2050.45 |
| Hainan | 3 | 3.26 | 181.67 |
| Hebei | 3.5 | 141.21 | 6806.44 |
| Henan | 3.4 | 137.05 | 2602.34 |
| Helongjiang | 2.7 | 52.19 | 642.58 |
| Hubei | 2.9 | 66.56 | 1424.46 |
| Hunan | 3.3 | 68.55 | 2214.68 |
| Jilin | 2.8 | 41.32 | 919.77 |
| Jiangsu | 2.2 | 105.38 | 334.56 |
| Jiangxi | 2.9 | 58.41 | 651.81 |
| Liaoning | 3 | 112.62 | 13394.29 |
| Nei Monggol | 3.7 | 140.94 | 7429.33 |
| Ningxia | 3.8 | 41.04 | 865.2 |
| Shandong | 3.1 | 182.74 | 1106.29 |
| Shanxi | 2.7 | 139.91 | 9187.25 |
| Shanxi(XIAN) | 3.8 | 91.68 | 1836.43 |
| Shanghai | 2.5 | 24.01 | 74.89 |
| Sichuan | 2.8 | 90.2 | 3988.2 |
| Tianjin | 2.6 | 23.09 | 9.15 |
| Xinjiang | 3.5 | 76.31 | 620.92 |
| Yunnan | 3.5 | 69.12 | 4969.03 |
| Zhejiang | 2.4 | 66.2 | 309.56 |
| Chongqing | 4.2 | 58.69 | 518.28 |
